# Stakeholders’ Perceptions Regarding Digital Therapeutics Reimbursement in South Korea: Qualitative Study

**DOI:** 10.2196/47407

**Published:** 2023-10-30

**Authors:** Boram Sim, Jin Han Ju, Byungsoo Kim, Jin Yong Lee

**Affiliations:** 1 HIRA Research Institute, Health Insurance Review and Assessment Service (HIRA), Wonju-Si, Republic of Korea; 2 Department of Drug Utilization Review (DUR), Health Insurance Review and Assessment Service (HIRA), Wonju-Si, Republic of Korea; 3 Department of Health Policy and Management, Seoul National University College of Medicine, Seoul, Republic of Korea; 4 Public Healthcare Center, Seoul National University Hospital, Seoul, Republic of Korea

**Keywords:** digital therapeutics, digital technology, mobile applications, internet-based intervention, reimbursement mechanism, qualitative research, perception, reimbursement, software, intervention, stakeholder, implementation, opinion, reimbursement method

## Abstract

**Background:**

Digital therapeutics (DTx) are therapeutic interventions driven by software and directly provided to patients, allowing them to manage their health with ease in any setting. A growing interest in DTx has spurred a discussion concerning their reimbursement pathways. However, DTx are still at a premature stage, with insufficient evidence on effectiveness, efficiency, and safety. Currently, although industries desire to quickly enter the market, especially by getting their products reimbursed by the National Health Insurance (NHI) fund, the NHI is cautious about DTx due to their uncertainties. Thus, public discussion and social consensus are crucial in deciding whether to reimburse DTx by the NHI fund.

**Objective:**

This study examined multiple stakeholders’ awareness and attitudes toward DTx and perceptions of regulatory pathways for adopting DTx.

**Methods:**

In-depth interviews were conducted with 11 stakeholders in South Korea (industry: n=4, health care: n=3, academia: n=2, and consumer: n=2) using semistructured guidelines. They were purposively sampled to identify individuals with expertise in DTx and NHI policies. The interviews were conducted either in person or via a videoconference for 45-70 minutes. Qualitative data were analyzed using directed content analysis, which uses interview guidelines as an analytical framework.

**Results:**

Findings were divided into three categories: (1) awareness and attitude toward DTx, (2) perception of whether DTx are worth entering the market and being reimbursed by the NHI fund, and (3) perception of how to enter the market and how to reimburse DTx by the NHI fund if they are worth it. Although consumer stakeholders were not familiar with the basic concept of DTx, the other stakeholders understood it thoroughly. However, all participants showed positive attitudes and acceptance of DTx. Most of them responded that DTx are worth entering the market, but they could not reach an agreement on the pathways for DTx to enter the market. Although participants were in favor of the reimbursement of DTx in principle, they responded that a conservative approach is required due to insufficient clinical evidence for DTx.

**Conclusions:**

We found that stakeholders in South Korea had positive attitudes toward DTx, perceived them as worth using, and agreed to allow them to enter the market. The main issue was not the problem of the technology itself but the difference in opinion as to the pathways for reimbursement. Therefore, this study concluded that the NHI fund, which is operated very conservatively, is insufficient to quickly adopt and implement DTx. Various reimbursement methods, including tax-based financing, raising innovation funds for new technologies, and pilot studies using the NHI fund, should be used to rapidly generate clinical evidence and reduce the uncertainties of DTx to secure a stable market.

## Introduction

Digital therapeutics (DTx) refer to software as a medical device that is independently operated from hardware. They are used to prevent, manage, and treat various illnesses [1]. The most acclaimed DTx product is the *reSET* mobile app developed by Pear Therapeutics. Approved by the US Food and Drug Administration in 2017, the *reSET* mobile app provides cognitive behavioral therapy for patients with substance use disorder. It has garnered global attention as studies proved its efficacy to treat diseases, unlike several previously launched health care apps [2]. Subsequently, DTx have gained momentum as a new treatment modality that may substitute or supplement conventional medical practices. Notably, as the COVID-19 pandemic prompted individuals to recognize the advantages of telemedicine, in which health care services could be provided remotely, the potential of DTx to deliver therapeutic interventions through software has gained significant attention [3]. Considering this recent growing interest in DTx, their market has been expanding rapidly [4,5].

The emergence of the DTx market has spurred discussions concerning reimbursement pathways, as securing them guarantees a stable market for DTx developers upon their release. In South Korea, where almost the majority of the population is included in the National Health Insurance (NHI), the fact of whether DTx is covered under the NHI has been directly linked to the future viability of DTx developers. However, the decision on whether medical technologies are eligible for reimbursement from the NHI requires careful consideration, given its implications on the population’s health and limited available financial resources. Thus, the clinical and economic aspects of medical technology are evaluated through several rounds of assessments after obtaining approval from the Korean Ministry of Food and Drug Safety (MFDS), including the New Health Technology Assessment (nHTA) and NHI benefit coverage determination. Reaching a final decision regarding these processes requires time, which can cause advanced technologies with short market cycles to abandon research and development due to delayed market entry. Thus, in March 2019, the South Korean government introduced a new track for the nHTA called Innovative Health Technology Assessment (IHTA), which allows technologies to collect evidence after rapidly entering the market, provided that it has substantial potential value. Moreover, some technologies designated as IHTA subjects can be reimbursed by the NHI temporarily. In November 2021, the South Korean government announced that they would consider DTx to be reimbursed via this track [6].

However, contrary to other innovative health technologies, DTx have not yet been launched in South Korea. Although 2 DTx for insomnia obtained approval from the MFDS in February and April 2023, they have not been released to the market yet. Furthermore, there are limited cases of development and use of DTx in overseas markets, which obscure their practicality. Moreover, it is challenging to ascertain whether physicians and patients will accept the novel technology. The therapeutic effectiveness of DTx hinges on sufficient patient engagement. Nevertheless, there is no guarantee that patients will consistently adhere to their treatment protocol [7,8]. Therefore, discussing DTx reimbursement currently constitutes a NHI fund investment with an uncertain future value. Hence, it is imperative to arrive at a consensus regarding how the principles of NHI reimbursement must be upheld and the opportunities that should be allocated to innovative health technologies. Nonetheless, most discussions are driven by only industry stakeholders, with no existing studies integrating diverse group opinions, including those of physicians and patients.

To our knowledge, the health care industry is urgently advocating for DTx reimbursement to secure a stable market after its development and establish a foothold for overseas expansion. Conversely, although the health care community acknowledges the potential of digital health technologies, there are increasing concerns that the value of DTx is currently overestimated [9,10]. Interestingly, in the academic community, some researchers are demanding appropriate regulatory reforms for these new technologies [11,12]. However, consumer perspectives are predominantly lacking in the existing body of literature. Therefore, this study aimed to examine diverse stakeholders’ awareness and attitudes toward DTx and their perceptions of regulatory pathways for adopting them. To the best of our knowledge, this is the first study to comprehensively analyze the perceptions of various stakeholder groups regarding DTx reimbursement in South Korea.

## Methods

### Overview

For this study, we conducted in-depth interviews to explore the stakeholders’ perceptions about DTx reimbursement. In-depth interviews are a qualitative research methodology wherein focused interviews are conducted with participants for an extensive exploration of their perspectives on a specific topic [13]. Thus, the interviews are conducted with a small sample size of participants, typically from 10 to 30 [13,14]. Data collection using individual interviews was deemed to be appropriate for this study, as the participants may have had varying levels of understanding about DTx and the health insurance reimbursement decision process.

### Ethical Considerations

This study was approved by the institutional review board of the Health Insurance Review and Assessment Service (2021-116-003). All participants were contacted by email or phone and agreed to be interviewed. They were reimbursed with KRW 200,000 (approximately US $ 148) for their participation in this study.

### Recruitment

In all, 11 academic, health care, industry, and consumer experts were included as participants to explore various group opinions using purposive sampling. Individuals who are currently developing DTx, have participated in their development process, or are conducting relevant research were included. We collected news published on this topic over the past year and identified experts with opinions on both DTx and NHI policies. Individuals who have participated in the NHI reimbursement decision-making were selected for the consumer group, as only a limited number of people have had exposure to DTx. Representatives of a civic group and a consumer group belonging to the Health Insurance Policy Deliberation Committee were selected for this study.

### Development of Interview Guidelines

We developed the interview guidelines following the latest government policy announcements [6]. Subsequently, these were finalized in collaboration with a qualitative research expert. The critical questions for the interview were about perception and attitude regarding DTx; whether it has high value to warrant market entry and reimbursement through the NHI fund; and if so, how they must be given market access (Table 1).

**Table 1.** Structure of the interview guidelines.

Categories	Questions
Perceptions about DTx^a^	Do you know what DTx are? What do you think the scope of DTx is? What benefits do you anticipate when DTx are adopted in health care? Conversely, what concerns do you have?
Perceptions about the reimbursement of DTx	Do you think that DTx should be covered by health insurance?
Perceptions about pathways to adopt DTx	Given that there is inadequate clinical evidence for DTx, what do you think about permitting the release of DTx products to the market before a health insurance coverage decision is made? In that case, what do you think about reimbursing it provisionally from the NHI^b^ fund (selective benefit^c^, where the NHI reimburses 10%)? What criteria should be used to assess products subject to the NHI reimbursement decision? Do you think it is appropriate to compare DTx with the standard of care that they are replacing or complementing? In this case, do you think that DTx could still be accepted based on their other benefits even if their effectiveness falls short of that of the standard of care?

a DTx: digital therapeutics.

b NHI: National Health Insurance.

c The selective benefit is a policy applying to medical services that do not have sufficient evidence yet, but the need for reimbursement is recognized. They are reimbursed by lowering the percentage paid by the National Health Insurance.

### Interview Procedures

The interviews were conducted either in person or via a videoconference from January 25 to February 15, 2022, by a female researcher (BS). Before the interview, the interviewer discussed the interview methods and contents with a qualitative research expert. The interview took place at a closed meeting room of Health Insurance Review and Assessment Service or at the participant’s workplace. Aside from the interviews with the 2 industry participants, nobody else was present during the interview apart from the participants and researchers. The 2 industry participants were accompanied by observers from their companies. There was no prior relationship between the interviewer and the participants.

The interview was conducted using semistructured guidelines. The interview topics were sent to the participants beforehand for them to organize their thoughts and opinions. They were also informed on the background and goals of the study, excluding the characteristics of the interviewer that could lead to bias. For the consumer group, the participants were asked whether they knew about DTx during the screening process, and it was found that DTx awareness was deficient among the participants. Thus, a brief explanation of the concept and overseas cases of DTx were provided to them.

The interviews lasted for 45-70 minutes, and the entire interview was recorded and transcribed. The researchers also took field notes during the interviews. All interviews were conducted in Korean and were subsequently translated to English for this paper. Interviews were conducted until saturation was reached.

### Analysis

This study performed a qualitative data analysis called directed content analysis, which involves analyzing data via a structured process using existing theories or study frameworks [15], using interview guidelines as the analytical framework.

For accuracy, the first author (BS), who conducted the interview, along with another researcher independently reviewed the interview recordings. After reading the recordings thoroughly to get an overall impression, the reviewers repeatedly read the transcriptions to grasp its meaning and highlighted the relevant statements. Furthermore, they made notes to identify critical themes and categorized them into predefined categories based on the guidelines. Unclassifiable content that featured a shared concept or category was established as a new category. For example, the perception of the reimbursement of DTx was divided into the perception of market entry and that of reimbursement by the NHI fund.

To improve the reliability and validity of the data analysis, the critical themes were repeatedly verified by 2 reviewers. Subsequently, all researchers discussed the content and names of the critical themes and arrived at a consensus on the primary findings. Additionally, we sought feedback on the findings and interpretations from experts with practical experience in health insurance reimbursement decisions not involved in this study. Finally, quotes were selected to illustrate each theme.

## Results

### Participant Characteristics

A total of 11 participants were included in this study (academia: n=2, industry: n=4, health care: n=2, and consumer: n=2). In all, 9 (82%) participants were male, and the participants were aged between their 30s to 60s (Table 2). The academia and health care groups were found to have experience and expertise on DTx. The industry group consisted of chief executive officers of companies that are in the progress of clinical trials approved by the MFDS. Thus, they had views and opinions on the adoption pathways of DTx. For the consumer group, even though they did not have experience related to DTx, they were well aware of the adoption pathways of new technologies in general.

**Table 2.** Participant profile.

Group and participant number		Sex	Age group (y)	Experiences
**Academia**				
	1	Male	30s	Experience in DTx^a^ policy development
	2	Male	50s	Experience in developing DTx product
**Industry**				
	3	Male	40s	CEO^b^ of a DTx company MFDS^c^-approved clinical trials in progress
	4	Male	30s	CEO of a DTx company MFDS-approved clinical trials in progress
	5	Male	50s	CEO of a DTx company MFDS-approved clinical trials in progress
	6	Male	50s	CEO of a DTx company MFDS-approved clinical trials in progress
**Health care**				
	7	Male	60s	Psychiatrist Experience in developing DTx product
	8	Male	40s	Psychiatrist Experience in developing DTx product
	9	Male	50s	Psychiatrist Experience in developing DTx product
**Consumer**				
	10	Female	60s	Representative of a consumer organization Experience on NHI’s^d^ benefit coverage determination
	11	Female	50s	Representative of a civil society Experience on NHI’s benefit coverage determination

a DTx: digital therapeutics.

b CEO: chief executive officer.

c MFDS: Ministry of Food and Drug Safety.

d NHI: National Health Insurance.

### Overview

**Figure 1.**
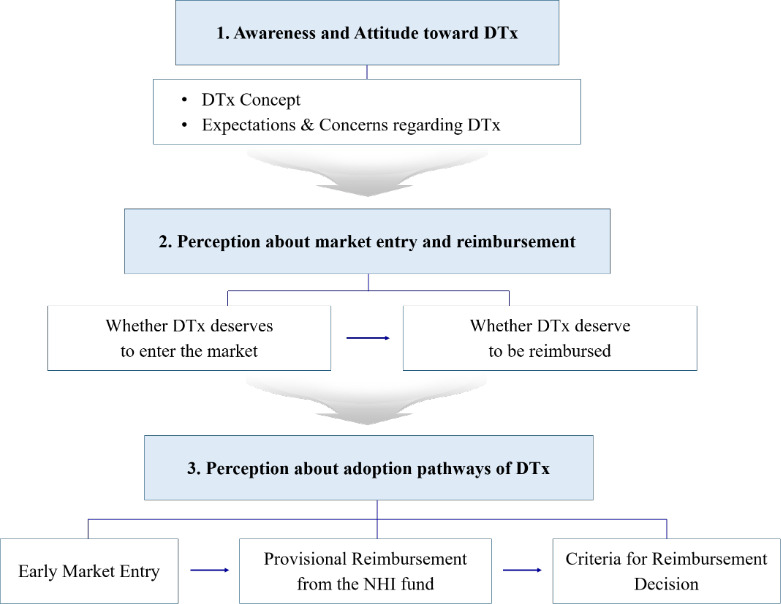
Framework of the study. DTx: digital therapeutics; NHI: National Health Insurance.

The findings were divided into three categories: (1) awareness and attitude toward DTx, (2) perception of whether DTx are worth entering the market and being reimbursed by the NHI fund, and (3) perception of how to enter the market and how to reimburse DTx by the NHI fund if they are worth it (Figure 1).

### Awareness and Attitudes Toward DTx

#### DTx Concept

**Figure 2.**
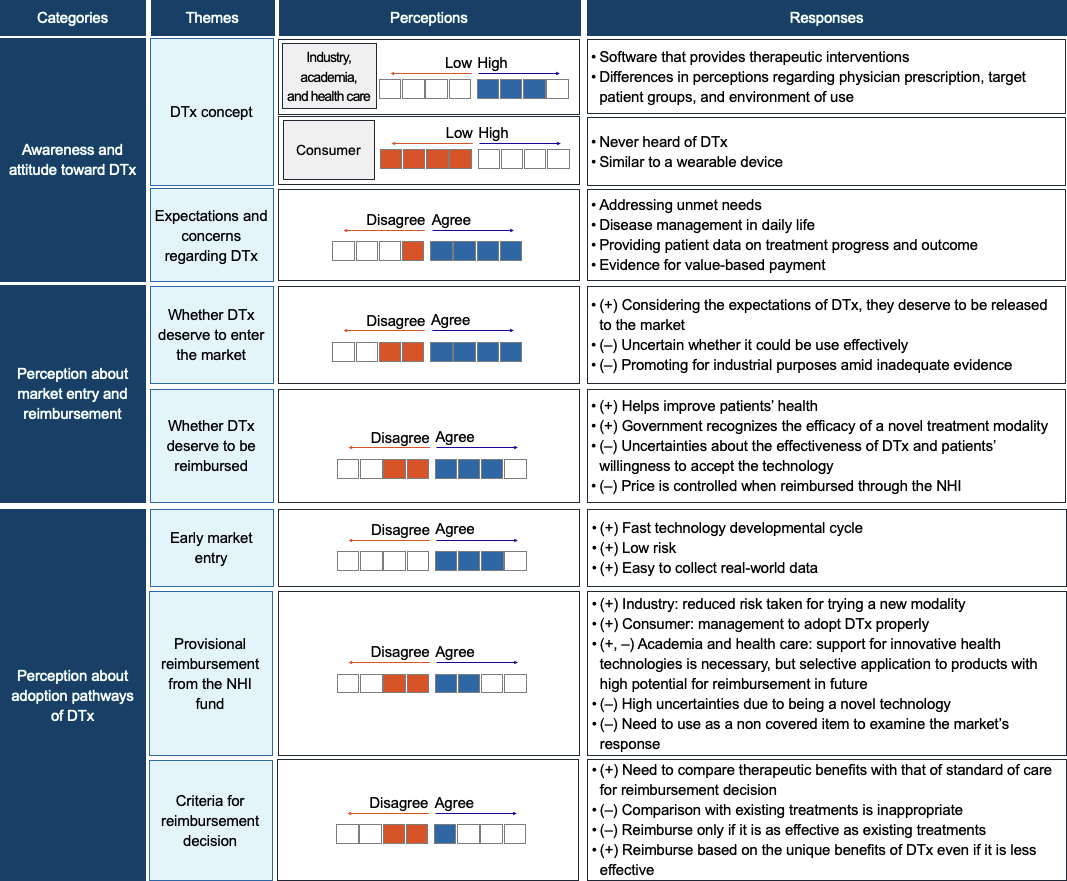
Awareness and perceptions about DTx and its market release and reimbursement. DTx: digital therapeutics; NHI: National Health Insurance.

It was found that all participants of the industrial, academic, and health care communities had a good understanding of the DTx concept as a “medical software that provides evidence-based therapeutic interventions” (Figure 2). However, the consumer group participants had limited knowledge of DTx. Participants with DTx knowledge emphasized that it must be distinguished from wellness products that aim to prevent the occurrence of diseases or maintain the well-being of people who are healthy or at health risk. Nevertheless, there were variances in their perceptions about its details, such as whether a physician’s prescription should be required, whether DTx include prevention before disease onset, and whether the places of product use should be differentiated (such as hospitals and patients’ homes).

Before, it was hard to come across information about DTx. So, I would say that my knowledge about DTx is just about the same as an average person’s. I have heard some explanations from a company that wanted to expand its business of a wearable [device] a while ago, so I have some basic understanding about it. However, when it comes to present-day DTx, I do not really know much about it.

[Consumer stakeholder, participant 10]

This is my first time learning about DTx. So, I searched the news before coming here.

[Consumer stakeholder, participant 11]

Just like the level of evidence differs between prescription drugs and health functional foods, there is bound to be confusion between DTx and healthcare products. It is crucial to differentiate them, especially in the early stages. The key to this is to have therapeutic evidence (clinical trials) just like prescription drugs.

[Academia stakeholder, participant 2]

The definition provided by the Digital Therapeutics Alliance does not actually mention the need for approval from the Korean Food and Drug Administration or a prescription from a doctor. Therefore, some [can] argue that we should perceive the category of DTx more broadly. In fact, in other countries, there is already a distinction between Prescription DTx (PDT) and Non-PDT.

[Health care stakeholder, participant 8]

It does not make sense to call anything that can be used like a general application as DTx. I think we should limit the scope of DTx to things that are actually prescribed by physicians and actually benefit patients when they use it.

[Health care stakeholder, participant 7]

Although there is no set business model for the DTx currently being developed, it could be used in hospitals or homes.

[Industry stakeholder, participant 6]

I personally think that anything prescribed by a doctor or applied to a patient should be considered DTx. When there is a prescription from a doctor, it could be applied both to the patient and to those who are at risk of disease. I believe that DTx should remain applicable to people in the pre-hypertension stage who does not need medication yet. If we treat people in the pre-hypertension stage, it prevents them from progressing to hypertension. Therefore, the scope of DTx should include prevention and management, not just treatment.

[Industry stakeholder, participant 5]

#### Expectations and Concerns Regarding DTx

The industry sector placed a high value on the fact that DTx can provide treatment that could not be adequately provided in the existing health care system. The industry experts specifically believed that DTx can reduce drug dependence by inducing behavioral changes and will ultimately reduce pharmaceutical spending and other societal costs eventually. Academic and health care experts emphasized that DTx help patients manage their condition in their daily lives and allows doctors to monitor the progression of treatment of their patients. Moreover, some expect DTx to create an opportunity for payers to pursue value-based care because they can collect treatment progress and outcome data through DTx. The consumer group participants also hoped that DTx would enable patients to assess and manage their conditions more objectively. As DTx provide a noninvasive therapeutic intervention, the participants expected the software to have few safety concerns.

To treat patients with insomnia properly, you need to use cognitive behavioral therapy, but it is underutilized in hospitals, right? DTx can fill this gap, providing proper treatment, reducing social costs, and improving the quality of life. It would have these kinds of benefits.

[Industry stakeholder, participant 5]

In the medical field, there are many gaps, especially in improving self-management and lifestyle habits to enhance health and overcome diseases in daily life. I think there could be a new paradigm shift in these areas.

[Health care stakeholder, participant 8]

Doctors also want to see patient data. I think DTx provides a reliable channel for doctors to access patient data, and through this, an agenda should be set on how the quality of care can be improved.

[Academia stakeholder, participant 1]

You know, we use several smartwatches these days. I think this is like a pre-DTx phase. This is why I think it is better from a consumer’s perspective because it clearly shows my status in numbers. But first, I think the patient must be willing.

[Consumer stakeholder, participant 11]

The Ministry of Food and Drug Safety (MFDS) strictly manages clinical trials and examines whether DTx are effective or harmful. However, they do that because they are worried that some treatments are going to be claimed effective, although they are not, not because there are safety issues.

[Health care stakeholder, participant 7]

### Perception on Whether DTx Deserve to Enter the Market

Although most participants perceived that DTx are suitable to be allowed market entry, they had concerns regarding their effective use. Specifically, they raised concerns about the “prescribing physicians’ lack of knowledge on using DTx,” “possible low therapeutic benefits from the inherent limitations of DTx,” and “uncertainty surrounding patient engagement.” Additionally, the consumer group participants expressed concerns regarding DTx use for commercial purposes.

DTx is a field where the necessity itself is clear. From the perspective of chronic patient management, it is certain that we will go in this direction in the future. There is no reason for not using it that has already been developed, and it can definitely be helpful in managing health. It would be great to introduce it quickly, but the problem is whether it has been validated and whether it really has that much effect.

[Health care stakeholder, participant 7]

When DTx is introduced, the level of education of prescribing physicians is one of the crucial factors that needs to be considered. There are some concerns that physicians may not be able to distinguish between general digital health devices and DTx and may not know how to use them properly and say things like, “How can I use this in treatment? How much do I need to use?”

[Health care stakeholder, participant 9]

Do older patients adapt well to DTx? Although companies developing and doctors prescribing these may be extremely enthusiastic, how actively will patients participate? Even after the pharmacist explains about a medicine thoroughly, the patients just decide not to follow the instruction the moment they turn away…I am also concerned that companies may push the product anyway despite predicting the side effects or results that fall short of expectations.

[Consumer stakeholder, participant 10]

### Perception on Whether DTx Deserve to Be Reimbursed

The participants responded that the reimbursement of DTx is necessary to improve patients’ health and enhance the financial stability of the NHI fund. However, some participants were cautious about reimbursement due to uncertainties about DTx’s effectiveness and acceptability. Nevertheless, the industry participants considered reimbursement important for securing a stable market and recognizing DTx to be distinct from general wellness products. Nonetheless, some industry participants state that they may choose different strategies that vary by product, because once a product gets reimbursed, its price will be controlled by the NHI, and it may be more advantageous as a noncovered item.

Wouldn’t reimbursement be needed if its effectiveness is proven and it is believed to be helpful for patients’ health?

[Industry stakeholder, participant 6]

Reimbursement from the NHI fund is necessary. I prefer HTA after its market release but if that’s difficult in our country, then I think market release after HTA is also good. (Reimbursement is needed) for sound finances of the NHI.

[Academia stakeholder, participant 1]

If the DTx is prescribed by a doctor in a hospital and used on a patient, then having it reimbursed allows more patients access to it, so we have that model in mind for now.

[Industry stakeholder, participant 5]

Having our product reimbursed is a particularly important issue for us because it means that the government acknowledges its effectiveness and officially compensates for it. This will help people distinguish therapeutics that they really need to use from health functional products they can just buy at a market.

[Industry stakeholder, participant 4]

It would be nice to have DTx reimbursed, but in terms of practicality, how willingly will the patients accept it?

[Consumer stakeholder, participant 11]

I’m a little cautious about the reimbursement. Reimbursing it as an independent therapeutic should be decided with more deliberation. This is the national budget we are talking about here, and I believe that if there is a better option with more concrete effectiveness, then it is right to give more money to that option.

[Health care stakeholder, participant 8]

From the company’s perspective, reimbursement is not necessarily all sunshine and rainbows because the price will be controlled by the NHI. It might be better to leave it as a non-covered item. So, I think I could say that companies do not necessarily want reimbursement of all DTx items they are developing.

[Industry stakeholder, participant 3]

### Perception on Adopting Pathways of DTx

#### Early Market Entry

Currently, DTx follow the IHTA track; hence, the products may be released in the market to accumulate clinical evidence before being subject to the nHTA [6]. The participants positively viewed approving its early market entry, considering the “rapid development cycle of DTx,” “low risk,” and “easier data collection for assessment.”

DTx is much easier to collect RWD (real-world data) from, and due to its digital nature, safety issues are of much less concern. This is why we are trying it on the field to collect data.

[Academia stakeholder, participant 2]

Considering the rapidly changing pace of technological development, we could keep falling behind if it takes too long to apply DTx to clinical settings.

[Industry stakeholder, participant 5]

Many companies are eager to enter the market early. The development is short, and the development cost is low, so even if they get MFDS approval, they are afraid that other companies will copy the form or algorithm before they are listed in the NHI.

[Health care stakeholder, participant 9]

#### Provisional Reimbursement From the NHI Fund

It was noted that the participants had conflicting opinions regarding the provisional reimbursement from the NHI fund for the DTx products with an early market entry. First, the industry group participants stated that provisional reimbursement is essential, considering that the NHI shares the risk taken by physicians and patients in trying a new treatment modality. The consumer group participants agreed to reimburse temporarily to alleviate patients’ financial burden and help effectively adopt DTx in the health care system. The academia and health care group participants considered provisional reimbursement positive for supporting the development of innovative technologies; however, they emphasized that products with potential must be adequately screened. Conversely, some participants stated that DTx should be released in the market as a noncovered item at first to examine the market’s reaction to this novel treatment modality.

Provisional reimbursement means that the government is sharing the risk, even if it is only 10%. I agree with that way from the perspective that it grants doctors some comfort when prescribing a new treatment.

[Industry stakeholder, participant 4]

If the purpose of the provisional reimbursement is to provide even the slightest support until they can show that the product is effective (I think it is needed), and of course, it would be great if all DTx products can be reimbursed, but if the financial resources do not allow it, then I think it would be right to apply it first to the products that have greater potential…Initiating reimbursement quickly may not be the best option. I hope a slower approach is taken so that both doctors and patients can experience the benefits of DTx through successful cases.

[Health care stakeholder, participant 8]

The concept is still relatively new and unfamiliar, so it may be necessary for it to start as a non-covered item to assess how much it is used. This is often the reason why non-covered items exist. However, from my viewpoint, regardless of whether it is provisionally reimbursed, it is not a big issue as long as it can be used.

[Health care stakeholder, participant 7]

I think it would be more rational to allow innovative medical technologies to enter the market quickly but with the price competitiveness being determined within the functions of users and suppliers. If it is effective, and there is demand among patients, they would be willing to pay for it, right?

[Industry stakeholder, participant 6]

#### Criteria for Reimbursement Decision of DTx

As the financial resources of the NHI are limited, new medical procedures, devices, or medicine are required to demonstrate their value through comparison with their alternatives. Most participants considered it to be suitable to compare the effectiveness of DTx with medical practices that they will substitute or complement, specifically in terms of the standard of care. However, there were conflicting opinions regarding the effectiveness level to be proven for DTx to be eligible for reimbursement. Some presented conservative opinions that suggested that DTx must have therapeutic benefits equivalent to an existing treatment to be eligible for NHI reimbursement. Others presented more modern perspectives, stating that even if DTx have lower therapeutic benefits than existing treatments, they should still be considered for reimbursement based on their unique benefits. The unique DTx benefits proposed were “increased access to treatment,” “improved patient experience and convenience,” “reduced adverse drug reactions,” and “lower societal costs.”

I think there should be a convincing criterion for reimbursement decisions. Of course, there may be criticism that the standard of care is not an equivalent comparison, but I do not think we should just see it that way, as there may be no other alternatives…

[Health care stakeholder, participant 8]

I think the basic condition is that even if the effectiveness is lower than expected, it should at least be as effective as the care originally provided in the hospital. However, if the difference in effectiveness is clinically acceptable, and there are other advantages like saving patients’ travel time, cost, and other social costs, we can consider reimbursement.

[Consumer stakeholder, participant 11]

If compared to treatment as usual (TAU), DTx would never be adopted. However, there are cases where DTx treatment is necessary, such as people who refuse to go to a mental health clinic, people who do not have time to go to a hospital, and so on, even if its effectiveness is somewhat lower.

[Academia stakeholder, participant 1]

I hope that DTx can be highly valued for its ability to provide treatments that are recommended in domestic and international clinical guidelines but are not actually available.

[Industry stakeholder, participant 4]

However, we should think about what level of effectiveness we are aiming for. There will surely be people who try to bring groundless things with absolutely no evidence for therapeutic potential and call it DTx.

[Health care stakeholder, participant 9]

## Discussion

### Principal Findings

The development of DTx is currently at an early stage globally; hence, their effectiveness, safety, efficiency, and other aspects have not been adequately proven. Thus, sufficient societal discussion and consensus are required to decide whether DTx deserve to be reimbursed through the NHI fund. As per our knowledge, this is the first study to explore various stakeholders’ opinions regarding the awareness and attitudes toward DTx and their perception on the regulatory pathways through which DTx should be adopted.

We found that the industry, academic, and health care experts possess a firm understanding of the fundamental concepts of DTx, whereas consumer experts have limited knowledge. Hence, it is highly probable that the general public remains entirely unfamiliar with DTx or has a limited understanding of them. This lack of awareness causes difficulty in accurately appraising the value of DTx [16]. Therefore, the public must be informed regarding what are DTx and their advantages and disadvantages to foster informed societal discourse.

It was found that all participants displayed favorable and receptive attitudes toward DTx. Most perceived that the DTx technology deserves market access and agreed to the quick adoption of this novel technology to prevent it from becoming obsolete. However, some participants expressed doubts about its clinical effectiveness, the appropriate use of the technology by health care providers, and patient engagement. In other words, the participants were optimistic about the transition in the health care system that DTx can bring and simultaneously recognized the high level of uncertainty associated with them. These findings align with prior research that cautioned to avoid overestimating DTx value without sufficient evidence [10,17].

This perception was also apparent in the participants’ attitudes toward the reimbursement of DTx. Notably, the industry experts sought reimbursement from the NHI fund to ensure a stable market, whereas the other stakeholders, despite recognizing the need for reimbursement, were cautious about the decision. Specifically, along with insufficient clinical evidence, a conservative approach is required rather than supporting lowering the financial burden of patients. Therefore, although several regulatory improvements to the market entry process were suggested, it is still crucial to ensure there is robust evidence to assess the eligibility of the technology for NHI coverage [18].

Nevertheless, the participants recognized the potential need for greater flexibility in the determination of reimbursement for DTx once the evidence of their effectiveness is established. This suggests that traditional criteria assessing clinical and economic value may not adequately capture the value of DTx. Notably, the participants of this study expected DTx to improve patient health and have wider benefits for the health care system, such as reducing unmet medical needs, lowering treatment and societal costs, and providing data on treatment progress and outcome. Therefore, going forward, the DTx value should be measured broadly and from multiple stakeholders’ perspectives, including those of patients, physicians, and payers, and the reimbursement criteria must reflect this broader DTx value perspective [19].

Taken together, this study highlights the necessity of an interim phase to address uncertainties regarding DTx, such as their therapeutic benefits and user acceptance, before DTx are covered by the NHI. Thus, it is reasonable to permit early market entry through the IHTA track but initially adopt DTx as a noncovered item to examine the market’s response to the technology. However, the South Korean government has announced its commitment to promoting the digital health care industry as a future growth engine [20], captivating several developers to delve into DTx development. Given the advanced information and communications technology infrastructure and medical technologies in South Korea, the country may produce globally competitive DTx products. Thus, the government must increase market predictability for these companies to develop quality DTx products. Various measures that reduce the financial burden of patients will facilitate the rapid introduction and expansive adoption of DTx technology. Several countries worldwide are using government subsidies or separate funds when introducing innovative technologies [21], which may provide some options for the South Korean government. Nevertheless, if DTx should be reimbursed by the NHI fund (or if the NHI is the best option for the reimbursement of DTx), it is necessary to clarify the purpose of paying through the NHI fund: to manage the use of DTx so that they are incorporated properly into the health care system, to accumulate robust real-world evidence that is acceptable for all stakeholders, and to contribute to the soundness of the NHI fund in long-term perspectives.

### Strengths and Limitations

This is the first study to comprehensively analyze the perceptions of various stakeholder groups on DTx reimbursement in South Korea. We succeeded in including diverse stakeholders of DTx in academic, industry, health care, and consumer experts who work on the front line of DTx and NHI policies; therefore, the information we collected was rich in content. However, these findings cannot be generalized to all stakeholders. Particularly, considering that this is an early stage of DTx, the participants in this study were mostly directly or indirectly related to DTx and may have greater expectations than the general population; thus, the results may seem to be favorable to the DTx industry. Therefore, the perceptions of a larger population pertaining to the reimbursement for DTx should be quantitatively explored after its widespread awareness. Nevertheless, this study is notable as a developmental study that extensively explored the DTx reimbursement issue and can be the basis for social consensus.

### Conclusions

The most important concern among stakeholders in South Korea was contrasting opinions regarding the pathway to reimbursement, as opposed to problems with the technology itself. The conservative NHI fund may be insufficient to quickly adopt and use this novel technology. Thus, concurrently using various pathways such as government subsidies and innovative funds for reimbursement to accumulate clinical evidence rapidly and eliminate uncertainties may be required to ensure that the technology does not fall behind in the market.

## Abbreviations

DTx: digital therapeutics
IHTA: Innovative Health Technology Assessment
MFDS: Ministry of Food and Drug Safety
NHI: National Health Insurance
nHTA: New Health Technology Assessment
